# Amputation totale du gland lors de la circoncision en milieu non hospitalier: rapport de cas

**DOI:** 10.11604/pamj.2022.42.214.28325

**Published:** 2022-07-19

**Authors:** Dimitri Kanyanda Nafatalewa, Augustin Kibonge Mukakala, Igor Mujinga wa Mujinga, Nasser Amisi Lubosha, Serge Ngoy Yumba, Manix Ilunga Banza, Vincent de Paul Kaoma, Prince Muteba Katambwa, Eric Mbuya Musapudi, Jeff Bukasa Misenga, Pitchou Mbey Mukaz

**Affiliations:** 1 Département de Chirurgie, Cliniques Universitaires de Lubumbashi, Faculté de Médecine, Université de Lubumbashi, Lubumbashi, République Démocratique du Congo,; 2 Département de Chirurgie, Cliniques Universitaires de Bukavu, Faculté de Médecine, Université Officielle de Bukavu, Bukavu, République Démocratique du Congo

**Keywords:** Circoncision, amputation, réimplantation du gland, cas clinique, Circumcision, amputation, glans reimplantation, case report

## Abstract

L´amputation du gland au cours de la circoncision est une complication tragique dont la responsabilité incombe à l´opérateur. Le traitement de référence de cette lésion repose sur la réimplantation microchirurgicale par anastomose vasculaire et nerveuse. Nous rapportons deux cas d´amputation totale du gland chez deux enfants: le premier âgé de cinq ans admis en urgence à la suite d´une circoncision et dont la réimplantation a été faite dans l´heure suivant l´accident, sans anastomose microchirurgicale; et le second était âgé de 11 ans reçu 3 ans après l´accident, géré psychologiquement jusque-là; il est en attente d´une chirurgie plastique. Le résultat obtenu après prise en charge du premier avait été jugé bon tant sur le plan urinaire, sur le plan de la sensibilité, de l´aspect cosmétique du gland et sur le plan érectile.

## Abstract (en)

Penile glans amputation during circumcision is a tragic operator-related complication. Standard treatment is based on microsurgical reimplantation with vascular and nerve anastomosis. We here report two cases of penile glans amputation in two children. The first patient was aged five years and was admitted urgently after circumcision. Penile glans was reimplanted without microsurgical anastomosis within one hour of the date of the accident. The other patient was aged 11 years and was received 3 years of the date of the accident. During these three years he had received psychological therapy. He was scheduled for plastic surgery. In the first patient, urinary, sensitivity and cosmetic results of the glans were good as well as erectile function.

## Introduction

La circoncision qui signifie excision en partie ou en totalité du prépuce est pratiquée depuis l´antiquité par certaines communautés surtout à des fins rituelles et initiatiques. C´est donc une intervention qui intéresse un organe dont les relations avec le psychisme sont très importantes [1]. Les complications de la circoncision sont nombreuses et certaines sont gravissimes pouvant engager le pronostic fonctionnel sexuel et urinaire, voire le pronostic vital, transformant ainsi un événement socialement heureux en un véritable drame familial [2]. L´amputation totale du gland lors de la circoncision est rare. Il s´agit d´un acte, aux conséquences incalculables et doit être considéré comme un véritable indicateur du degré d´incivisme à l´heure où la circoncision représente l´intervention chirurgicale la plus réalisée au monde [3], réimplantation balanique après accident de circoncision et [4], amputation du gland lors de la circoncision: à propos de 19 cas. Elle est vécue comme un véritable drame non seulement en raison de l´importance de la verge mais également en raison de la simplicité de la circoncision qui n´est toutefois pas dénuée de risque [1,2]. Dans les pays développés [1,5], le taux de complications varie de 2 à 5%. Ce taux est plus élevé dans les pays en voie de développement [2,6] et peut atteindre jusqu´à 85% quand le geste est réalisé par des circonciseurs traditionnels. De nos jours, le traitement standard de cette complication repose sur une réimplantation microvasculaire et nerveuse, ce qui garantit la fonction érectile, la fonction urinaire et la sensibilité de l´organe amputé. Cependant, la circoncision étant l´intervention chirurgicale la plus pratiquée dans le monde, il est évident que dans de nombreux pays en particulier en développement, les plateaux techniques n´offrent pas les conditions pour une telle réimplantation microchirurgicale. Par conséquent, la seule alternative reste la réimplantation sans anastomose micro vasculaire et nerveuse avec les risques de fistule, de nécrose, de sténose, de troubles érectile et de la sensibilité du gland [7,8]. Nous rapportons deux observations d´amputation totale du gland survenue en milieu non hospitalier: le premier à l´âge de 5 ans et le second âgé de 11 ans tous, prise en charge dans notre service.

## Patient et observation

### Observation I

**Figure 1:**
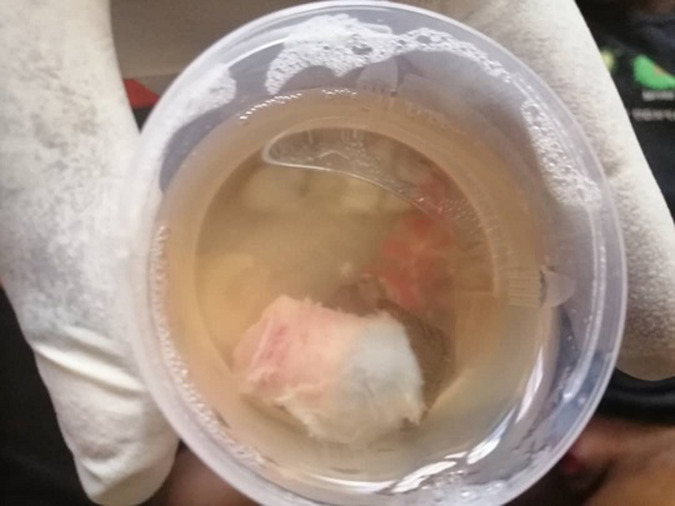
prépuce et gland amputés et conservés dans le sérum physiologique

**Présentation du patient**: un patient âgé de 5 ans, sans antécédents pathologiques particuliers était amené en consultation en urgence pour amputation du gland survenue au décours d´une circoncision réalisée 1h30´ avant, par un infirmier à domicile. La section complète du gland a été immédiatement reconnue, et le malade avait été acheminé dans nos services pour une prise en charge avec le gland et le prépuce excisé placé dans un flacon de sérum physiologique (Figure 1).

**Figure 2:**
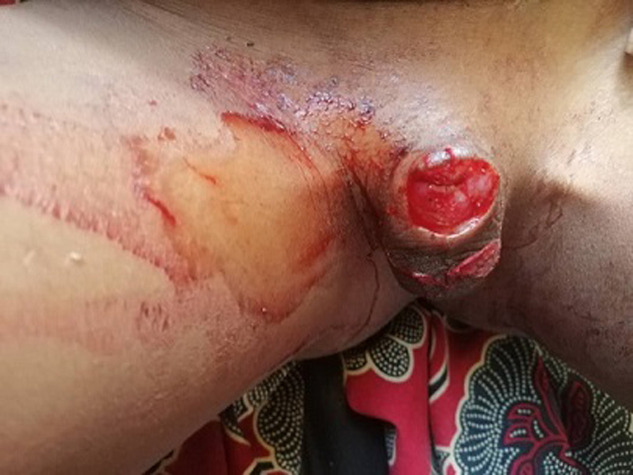
moignon du pénis après amputation traumatique du gland avec l’orifice de l’urètre visible sur la tranche de section

**Résultats cliniques**: à l´examen clinique, son état général était marqué par l´agitation, le petit enfant présentait des pleurs incessants. Ses conjonctives palpébrales étaient colorées, les bulbaires anictériques, la fréquence respiratoire était de 32 cycles/minute, la fréquence cardiaque de 98 battements/minute. L´examen des organes génitaux avait révélé des garnitures souillées par du sang rouge vif avec par endroit des caillots sanguin, à l´ablation desquelles était observé un moignon du pénis d´environ 2 cm de long et 2,5 cm de circonférence saignant activement au niveau de la tranche de section qui était plane et à bords nets (Figure 2).

**Démarche diagnostique**: au vu de ces éléments le diagnostic d´amputation accidentelle du gland compliquée d´une anémie tolérée était confirmée et le patient avait été rapidement amené au bloc opératoire après avoir déterminé le groupage sanguin, le taux d´hémoglobine, d´hématocrite, dont les résultats avaient indiqué une transfusion d´une unité de sang total isogroupe, isorhésus, et sérocompatible en urgence.

**Figure 3:**
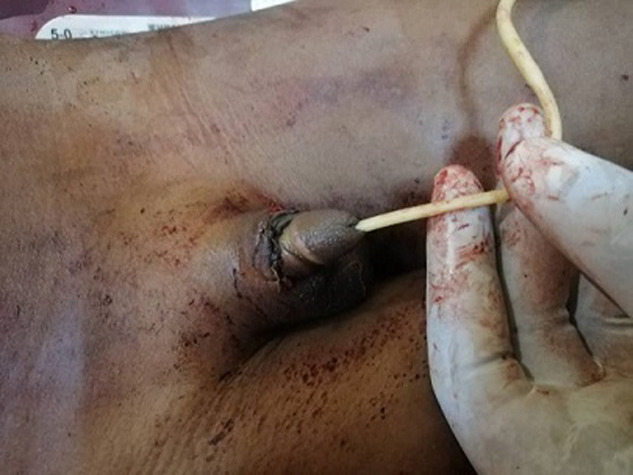
gland réimplanté avec une sonde urinaire en place

**Intervention thérapeutique**: après la visite pré-anesthésique, la patient avait été classée ASA I et nous avions procédé au lavage du gland et du prépuce amputés au sérum physiologique tiède. La réimplantation du gland avait été réalisée sans microscope par rattachement du gland avec anastomose (Figure 3) en deux plans: le corps spongieux et la couche périphérique de tissu érectile après la pose d´une sonde tutrice type Foley charnière 10. L´immobilisation a été obtenue par un pansement non compressif. La durée écoulée entre l´amputation et le début de l´intervention réparatrice était d´une heure et demie.

**Figure 4:**
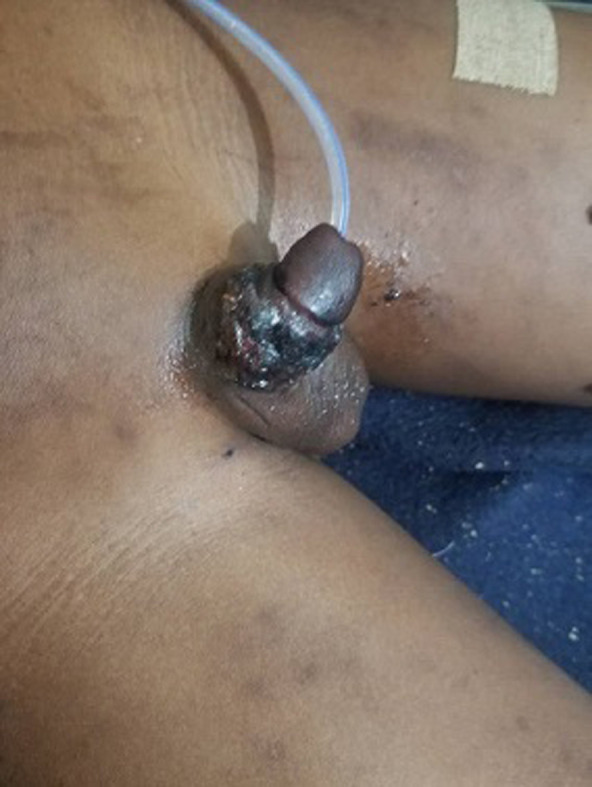
pénis au neuvième jour post réimplantation et remplacement de la sonde urinaire

**Figure 5:**
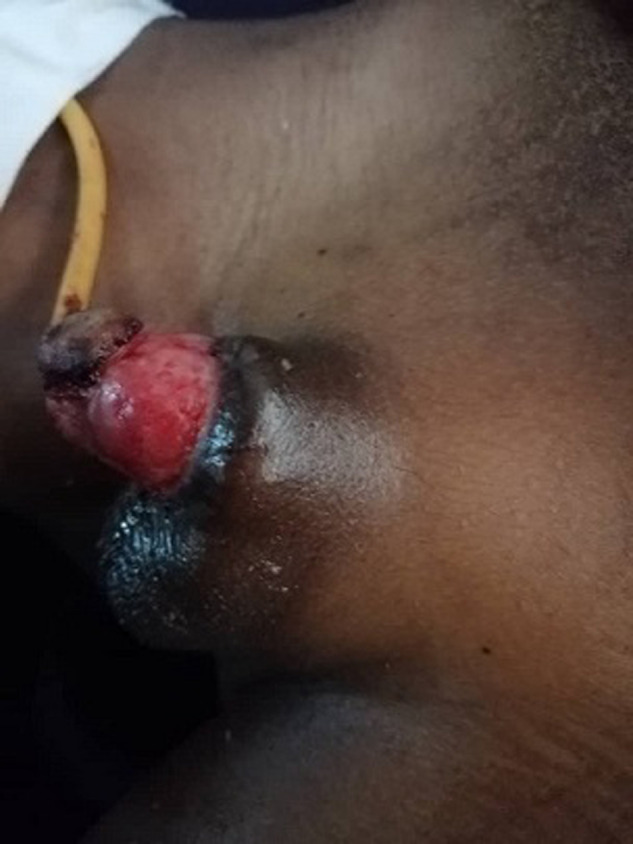
40^e^ jour après la réimplantation du gland

**Figure 6:**
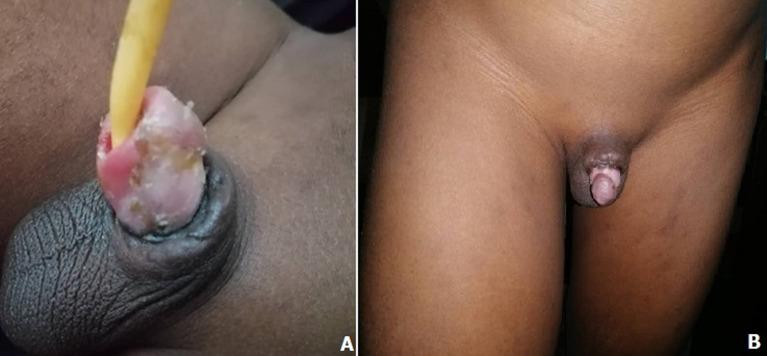
A) 55^e^ jour post réimplantation du gland; B) 90^e^ jour post réimplantation et après ablation de la sonde urinaire

**Suivi et résultat de l´intervention thérapeutique**: les soins post opératoires ont consisté en une analgésie, une sérothérapie antitétanique, une antibiothérapie parentérale et une application locale du mercurochrome. Le pansement avait été changé au J7 et la sonde urinaire avait été changée au J14 montrant un gland de coloration normale et saignant à la piqûre (Figure 4). Le pansement du 40^e^ jour montre un pénis de coloration rosée, sensible, saignant à la piqûre avec une sonde urinaire en place (Figure 5). Au 55^e^ jour, le pénis est de coloration rosée, conservant sa sensibilité, érectile, et saignant à la piqûre (Figure 6A). La sonde urinaire a été enlevée au 90^e^ jour soit trois mois après la réimplantation (Figure 6B).

**Consentement éclairé**: nous avons obtenu pour la rédaction et la publication de ce travail, un consentement éclairé et signé du patient.

### Observation II

**Présentation du patient**: il s´agit d´un patient âgé de 11 ans, amené en consultation par ses parents 3 années après l´amputation totale du gland survenue lors de la circoncision à domicile quand celui-ci avait 8 ans d´âge. Il avait été pris en charge dans un centre hospitalier périphérique ou une hémostase avait été faite sans tentative de réimplantation du gland par manque de ressources. L´évolution avait été marquée par la cicatrisation du moignon.

**Figure 7:**
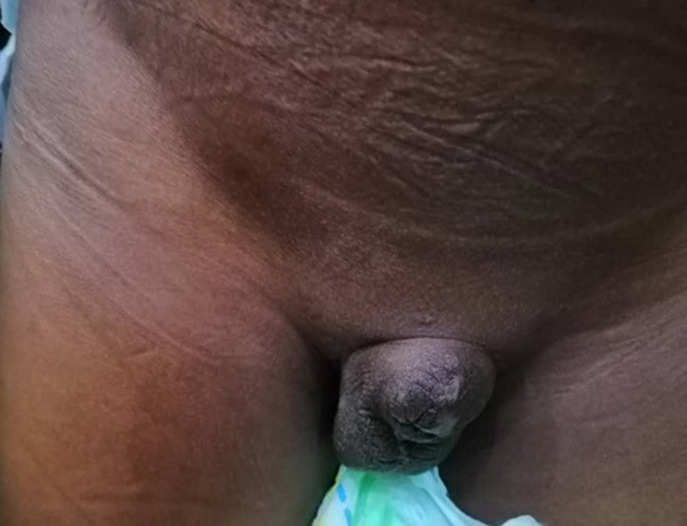
moignon du pénis avec la tranche de section déjà cicatrisée et l’orifice urétral perméable

**Résultats cliniques**: à notre examen, on note l´absence du gland, le moignon de la verge mesure environ 2,5 cm de longueur et 3,5cm de diamètre à la base. L´urètre était perméable, à bord net et déjà cicatrisé (Figure 7).

**Intervention thérapeutique**: aucune prise en charge chirurgicale n´a été instaurée et l´on s´était contentait d´une prise en charge psychologique de l´enfant et de son entourage.

**Suivi et résultat de l´intervention thérapeutique**: après un consentement libre des parents, le malade a été libéré avec programme de le revoir à l´âge de 18 ans pour une éventuelle pose de prothèse.

**Consentement éclairé**: après échanges avec l´équipe médicale, les parents de nos patients avaient consentis librement aux protocoles thérapeutiques et avaient exprimé l´avis favorable au programme établi.

## Discussion

La verge est un organe masculin doté de double fonctions: une fonction urinaire et une fonction de copulation. La circoncision est intervention chirurgicale largement pratiquée de par le monde dans un but religieux, coutumier ou pour des raisons médicales [9]. Dans de nombreuses communautés, en particulier en Afrique, au Sud du Sahara où la circoncision est pratiquée de façon courante, elle a encore un statut de petite chirurgie. Cela laisse libre cours aussi bien aux paramédicaux qu´à des non-professionnels de la santé à sa pratique dans des conditions non conformes moyennant le payement « d´honoraires ». Étant de loin l´intervention chirurgicale la plus pratiquée dans le monde, aussi bien pour des motifs religieux, rituels, socioculturels, esthétiques que médicaux, la circoncision peut être source de complications nombreuses. Les complications hémorragiques sont les plus fréquentes [3,10]. Elles se manifestent sous forme de saignement ou d´hématome. Elles sont dues à un défaut ou un trouble de l´hémostase. Une plaie du gland, une lésion de l´artère du frein ou même l´hémophilie peuvent en être la cause. Habituellement, ces complications hémorragiques sont facilement jugulées par un pansement compressif; parfois, une exploration de la plaie s´avère nécessaire pour assurer l´hémostase. Dans le cas de l´hémophilie, la prise en charge du patient nécessite un apport de facteurs de coagulation dont il manque, en particulier les facteurs VIII et IX. L´infection vient au deuxième rang après les complications hémorragiques [11,12]. L´amputation de la verge est rare et survient lors de la circoncision, d´un acte criminel, ou une automutilation.

Dans les pays développés [1,13], le taux de complications varie de 2 à 5%. Ce taux est plus élevé dans les pays en voie de développement [2,4] et peut atteindre jusqu´à 85% quand le geste est réalisé par des circonciseurs traditionnels. Dans les pays d´Afrique du Nord, ce type d´accident touche essentiellement l´enfant au cours d´une circoncision [4]. Dans notre milieu nous n´avons retrouvé aucune étude antérieure abordant ce sujet. Nos patients étaient jeunes: 5 ans et 8 ans au moment des faits. Sherman *et al*. [12], et Shaeer *et al*. [13] dans leurs séries rapportent que l´âge moyen des patients était plus bas aux USA et que la circoncision est le plus souvent réalisée en période néonatale. La prise en charge thérapeutique dépend essentiellement du délai de consultation. Dans notre cas le premier a consulté une heure 30 minutes après l´accident et le second 3 ans après. [4] ont réalisé une réimplantation, avec succès, après environ 150 minutes d´ischémie. Gluckman *et al*. [11] rapportent un bon résultat après réimplantation par anastomose urétrale et glandulaire en s´aidant d´une loupe grossissante chez un nouveau-né reçus 3 heures après l´accident. Sherman *et al*. [12] rapportent dans leurs séries un succès après réimplantation jusqu´à la huitième heure après l´accident et cela après simple anastomose du tissu glandulaire ampute. D´autres auteurs, après réimplantation microchirurgicale du gland, rapportent de bons résultats après 16 heures [14] voire 18 heures [8,13] d´ischémie. Certains proposent même une réimplantation jusqu´à la 24^e^ heure [2]. Nous pensons donc qu´il est nécessaire de tenter une réimplantation chaque fois que possible devant un tel cas, et lorsque la partie amputée est de bonne qualité. Le tissu amputé est au mieux transporté dans un flacon stérile refroidi sans contact direct avec la glace qui peut ischémier les tissus [6]. Dans notre cas, le tissu amputé a été transporté dans un flacon de sérum physiologique. Ceci a été aussi rapporté dans l´étude menée par Essid *et al*. [3] en Tunisie.

La technique de réimplantation recommande de traitée selon les principes standards de greffe de la chirurgie reconstructrice sous une technique microvasculaire, en s´aidant d´une loupe grossissante. Pour plus de facilité, l´intervention commence par l´anastomose urétrale sur sonde tutrice utilisant un fil mono filament à résorption lente 5/0 ou 6/0. L´anastomose du corps spongieux va assurer la stabilité du champ opératoire et permettre la réalisation des autres sutures sans tension. Les greffes de peau libre représentent le traitement de choix afin d´obtenir le minimum de cicatrice rétractile [3,7,8,11-13]. On réalise également une anastomose termino-terminale de l´urètre, du corps spongieux et du corps caverneux, ce geste étant complété par des anastomoses vasculaires et nerveuses réalisées sous microscopie opératoire. Dans le cas où la prise en charge est tardive comme c´est le cas du deuxième patient [14]; on recommande un remplacement par greffe de muqueuse libre, utilisant surtout la muqueuse buccale. Ce n´est que lors de l´échec ou de l´impossibilité de cette technique qu´on sera amené à tenter une phalloplastie des corps caverneux désinsérer des branches ischio-pubiennes. Dans le premier cas, le traitement a consisté à un débridement de moignon de la verge et du gland, à la pose de la sonde par le méat du gland amputé puis dans l´urètre pénien, l´anastomose des corps spongieux et la suture cutanée à l´aide du vicryl 3/0 en points séparé. La réimplantation sans anastomose microchirurgicale est possible mais pourvoyeuse de séquelles (nécrose cutanée, sténose urétrale [13].

La sonde urétrale doit être gardée, habituellement dans notre école jusqu´au 21^e^ jour, pour assurer une bonne prise du tissu greffé et de prévenir la sténose cicatricielle du méat urétral. Essid *et al*. [3] recommande de la garder entre 20 et 30 jours. Vu l´apport sanguin relativement pauvre des tissus amputés, l´antibiothérapie est indiquée pour prévenir l´infection [3]. Dans notre cas, le premier patient était soumis à une forte antibiothérapie (bithérapie associant une céphalosporine et un imidazole). La sténose urétrale constitue la complication post-opératoire la plus fréquente nécessitant des dilatations ou une révision.

## Conclusion

La circoncision n´est pas une intervention chirurgicale banale et doit être pratiquée dans une formation médicale bien équipée et cela par un médecin qualifié. L´amputation du gland lors de la circoncision est rare. Elle doit attirer l´attention de tout médecin et devra être reconnue aussitôt. La prise en charge doit être multidisciplinaire faisant appel à diverses spécialistes: chirurgien urologue, andrologue, psychiatre, chirurgien esthéticien et plasticien. La réimplantation doit être tentée chaque fois que possible. L´évolution dépend de la précocité du diagnostic et du traitement, de la qualité du gland amputé, et de la technique utilisée.
